# Measles and Scarlet Fever

**Published:** 1875-07

**Authors:** E. H. Gibbs


### MEASLES AND SCARLET FEVER.

BY E. H. GIBBS, A.M. M.D.

Mistakes frequently occur in confounding these diseases—which in many respects resemble each other—and it is important to be able to readily distinguish them. Not so important in view of the medical treatment, as in the care the patient should receive.

The most marked symtoms of MEASLES are: aching in the limbs, chilliness and lassitude, followed by a dry skin, furred tongue and other indications of fever. With these symptoms—or sometimes previous to them—there is irritation of the mucuous membrane of the eyes, nose and mouth, with sneezing, discharge of tears, sore throat, dry cough and on the second or third day, considerable redness of the soft palate and tongue. The rash usually appears on the fourth day, commencing on the face and neck and extending over the rest of the body. This rash occurs in small, red, distinct spots, like flea-bites, and appearing in groups. The disease begins to subside about the eighth day.

SCARLET FEVER

is characterized by an aggrevation of some the above symptoms. The fever sets in with a rapid pulse, dry, hot skin, flushed face, furred tongue, thirst and great weakness, and generally vomiting, headache, restlessness, delirium and frequently convulsions. The throat, mouth and tongue are inflamed and swollen. The tongue is covered with a yellowish-white fur, with projecting red papillæ upon its surface. The tip and edges of the tongue are red.

The rash usually appears on the second day; commencing on the chest and arms, and diffusing itself over the body in about twenty-five hours, covering it with a dark red hue. The surface is smooth to the touch, the rash not being perceptibly elevated. There are, however, exceptions to this rule. The fever does not abate upon the appearance of the rash, but continues with various degrees of violence throughout its whole progress. The disease attains its height from the fifth to the ninth day, when *desquamation* begins. This is often attended with itching and tenderness of the skin. The process of *desquamation* is assisted and the comfort of the patient greatly promoted by annointing the entire body with sweet oil, or what is probably better and more convenient, with a piece of fat bacon or ham. It softens the cuticle and allays the irritation, and consequently there will be a marked improvement in the other symtoms.

SCARLET RASH.

This is a rose-colored efflorescence without pimples. It is not contagious. The affection often causes considerable solicitude on account of of its resemblance to the first stage of scarletina. It requires no treatment. It is only intended to describe the more prominent symptoms of these diseases, without reference to the great variety in form and intensity.

Nearly all have measles, sooner or later. No disease requires more careful attention. The patient must be protected against sudden changes in temperature and from draughts of air. Little treatment is required. It is now generally admitted that Scarlet Fever is a contagion that it is possible to avoid until the age of twelve or fifteen years, when it is not likely to be contracted. How to avoid it then, is what most concerns us. This can be done to an extent that will make the disease of comparatively rare occurance. It is probably within bounds to say that one third of the children who are exposed do not take it. There must be some cause for this exemption, and some reason why the other two-thirds so exposed are certain to contract the disease. The same rule applies in this as in other diseases. It is the strong and robust who keep the systom toned up by sufficint food and rest, who are cleanly and temperate, that resist disease in all its forms. It is those who disregard these things that become victims to disease.

But, it will be truly said, that many fine, healthy children contract Scarlet Fever.

This is probably because at the moment they are exposed to the disease the vital tone of the system is temporarily lowered from fatigue, hunger or want of sleep. These views are not merely theoretical; they have been confirmed by years of careful observation. Assuming them to be correct, the course to be pursued with children, in order to protect them from contagious and other diseases, is to keep them well fed, and clean. Let them have sufficient and regular exercise in the open air, but not to an extent to produce great fatigue. When they are unwell, particularily if there is loss of appetite, keep them at home until they are well again. Many a mother can date the illness of a child from the day it was sent to school, sick and weary, and in condition to contract disease with the first breath of contaminated air inhaled.

---

When the heaven is black with storm and the sun is hid, how hideous are the clouds, but when the clouds are moving on, and the sun gets a full stroke at them, making them glow with all colors from the deepest to the faintest, where are the castellated battlements so beautiful; what in the earth or sky is there so magnificent.

---

We are all a kind of chameleon, taking one hue, the hue of our moral character, from those who are about us.

---

Great wants proceed from great wealth, but they are undutiful children, for they sink wealth down to poverty.

---

Idlers cannot even find time to be idle, or the industrious to be at leisure. We must be always doing or suffering.

---

He who wishes to travel far is careful of his steed; drink, eat, sleep, and let us burn a fire which shall continue to burn.

---

All flowers will droop in absence of the sun that waked their sweets.

